# rTMS Induces Brain Functional and Structural Alternations in Schizophrenia Patient With Auditory Verbal Hallucination

**DOI:** 10.3389/fnins.2021.722894

**Published:** 2021-09-01

**Authors:** Yuanjun Xie, Muzhen Guan, Zhongheng Wang, Zhujing Ma, Huaning Wang, Peng Fang, Hong Yin

**Affiliations:** ^1^Department of Radiology, Xijing Hospital, Fourth Military Medical University, Xi’an, China; ^2^Department of Mental Health, Xi’an Medical University, Xi’an, China; ^3^Department of Psychiatry, Xijing Hospital, Fourth Military Medical University, Xi’an, China; ^4^Department of Clinical Psychology, School of Medical Psychology, Fourth Military Medical University, Xi’an, China; ^5^Department of Military Medical Psychology, School of Medical Psychology, Fourth Military Medical University, Xi’an, China

**Keywords:** schizophrenia, auditory verbal hallucination, transcranial magnetic stimulation, MCCB, amplitude of low-frequency fluctuation, voxel-based morphometry

## Abstract

**Background:**

Low-frequency transcranial magnetic stimulation (rTMS) over the left temporoparietal cortex reduces the auditory verbal hallucination (AVH) in schizophrenia. However, the underlying neural basis of the rTMS treatment effect for schizophrenia remains not well understood. This study investigates the rTMS induced brain functional and structural alternations and their associations with clinical as well as neurocognitive profiles in schizophrenia patients with AVH.

**Methods:**

Thirty schizophrenia patients with AVH and thirty-three matched healthy controls were enrolled. The patients were administered by 15 days of 1 Hz rTMS delivering to the left temporoparietal junction (TPJ) area. Clinical symptoms and neurocognitive measurements were assessed at pre- and post-rTMS treatment. The functional (amplitude of low-frequency fluctuation, ALFF) and structural (gray matter volume, GMV) alternations were compared, and they were then used to related to the clinical and neurocognitive measurements after rTMS treatment.

**Results:**

The results showed that the positive symptoms, including AVH, were relieved, and certain neurocognitive measurements, including visual learning (VisLearn) and verbal learning (VerbLearn), were improved after the rTMS treatment in the patient group. Furthermore, the rTMS treatment induced brain functional and structural alternations in patients, such as enhanced ALFF in the left superior frontal gyrus and larger GMV in the right inferior temporal cortex. The baseline ALFF and GMV values in certain brain areas (e.g., the inferior parietal lobule and superior temporal gyrus) could be associated with the clinical symptoms (e.g., positive symptoms) and neurocognitive performances (e.g., VerbLearn and VisLearn) after rTMS treatment in patients.

**Conclusion:**

The low-frequency rTMS over the left TPJ area is an efficacious treatment for schizophrenia patients with AVH and could selectively modulate the neural basis underlying psychiatric symptoms and neurocognitive domains in schizophrenia.

## Introduction

Schizophrenia is a severe and chronic mental disorder that is characterized by diverse psychopathology, including positive symptoms, negative symptoms, and cognitive impairments (Os and Kapur, 2009). Auditory verbal hallucinations (AVH), erroneous perceptions of voices in the absence of external stimuli, are among the main symptoms of schizophrenia reported by 50–70% of patients with schizophrenia (Llorca et al., 2016). Neuroimaging studies have demonstrated that AVH is associated with increased activity in brain regions involving speech perception, including left and right superior temporal cortex (Lennox et al., 2000; Shergill et al., 2000a), left temporoparietal cortex (Silbersweig et al., 1995), and Broca area (McGuire et al., 1993), which reflect abnormal activation of normal auditory pathways. It has been demonstrated that low-frequency (e.g., 1 Hz) repetitive transcranial magnetic stimulation (rTMS) produces a sustained reduction in brain activation that directly stimulated and other relevant brain areas (Boroojerdi et al., 2000; Rossi et al., 2000) and suggests the decreased excitability of pyramidal neurons and long-term depression-like neuroplasticity changes (Touge et al., 2001; Hoffman and Cavus, 2002).

Consequently, low-frequency rTMS delivering to brain regions critical to auditory speech perception may reduce the severity of AVH in schizophrenia. The left temporoparietal junction (TPJ) area appears the most optimal target region for low-frequency rTMS treatment in schizophrenia. Many studies have investigated the efficacy of this setup and have indicated that low-frequency rTMS patterns over the left TPJ area significantly decreased the AVH severity of schizophrenia (Tranulis et al., 2008; Demeulemeester et al., 2012; Otani et al., 2015). Meanwhile, cognitive impairments, such as deficits in working memory (WM), attention, executive function, and processing speed, are core features of schizophrenia (Bora, 2015; Lystad et al., 2017), occurring in a different stage of this disease. Although high-frequency rTMS has been evidence for reducing cognitive impairments (Barr et al., 2013; Jiang et al., 2019; Xiu et al., 2020), low-frequency rTMS also significantly improves certain cognitive functions in schizophrenia (Brunelin et al., 2006; Hoffman et al., 2013). However, the previous studies have shown that the effect of low-frequency rTMS on AVH and cognition in schizophrenia was mixed (Schneider et al., 2008; Lage et al., 2016; Marzouk et al., 2020).

Hence, it is necessary to understand the underlying neural substrate of the rTMS treatment effect to decide whether it is a productive approach for treating schizophrenia. Recently, resting-state functional magnetic resonance imaging (fMRI) has become a promising tool for exploring brain activity and enhancing our understanding of the pathophysiology of schizophrenia (Bassett et al., 2012; Rubinov and Bullmore, 2013). Both functional and structural abnormalities have been reported in schizophrenia by resting-state fMRI data. In the functional domain, the abnormal amplitude of low-frequency fluctuation (ALFF) has been observed in the temporal, occipital, cingulate cortex, and subcortical structures (e.g., hippocampus and thalamus; Alonso-Solis et al., 2017; Hare et al., 2017). For instance, schizophrenia patients with AVH showed increased ALFF in the bilateral temporal role and parahippocampus gyrus, while decreased ALFF in the parietal, occipital, and cingulate cortex. In the structural domain, smaller gray matter volume (GMV) with AVH in schizophrenia patients, as measured with voxel-based morphometry (VBM), have been identified in a wide range of brain regions, including the prefrontal, parietal, temporal, and occipital cortex (Dean et al., 2020; Zhuo et al., 2020).

Few studies have investigated the impact of rTMS treatment on brain activation for schizophrenia patients with AVH. In a first preliminary study, Fitzgerald et al. (2007) reported increased task-related activation in brain areas involved in speech processing circuity (e.g., left inferior frontal gyrus and angular gyrus) in schizophrenia patients with refractory hallucinations after rTMS treatment. In a PET and low-resolution brain electromagnetic tomography (LORETA) study, Horacek et al. (2007) found that schizophrenia patients with auditory hallucinations showed decreased brain metabolism in the left superior temporal gyrus and increased metabolism in the contralateral cortex when low-frequency rTMS was applied to the left temporoparietal cortex. A similar PET study indicated that rTMS-treatment schizophrenia patients with AVH had reduced neuronal activity in language-related regions (e.g., primary auditory and left Broca area; Kindler et al., 2013). Recently, using an inner speech task, Bais et al. (2017) evaluated the effect of rTMS treatment on task-related brain networks in patients with schizophrenia and AVH. They observed that rTMS over the left TPJ area resulted in deactivation of the left supramarginal gyrus and bilateral frontotemporal network, reducing the likelihood of speech intrusion. These results suggest that rTMS over the left TPJ area may affect neural activation in relevant regions for schizophrenia patients.

Although low-frequency rTMS has been applied to treat schizophrenia patients, the underlying neural mechanisms through which rTMS exert such therapeutic effect remain inadequately understood. The present study investigated the effect of 1 Hz rTMS stimulation over the left TPJ area in schizophrenia patients with AVH and identified functional (ALFF) and structural (GMV) alternations using resting-state (fMRI) data. A battery of clinical and neuropsychological assessments was collected and used to evaluate whether the brain alternations were associated with clinical and neurocognitive profiles following the rTMS treatment in patients.

## Materials and Methods

### Subjects

Patients met diagnostic criteria for schizophrenia using the Structural Clinical Interview for DSM-V (Stein et al., 2010). The main inclusion criteria were medication-resistant auditory hallucinations for at least one conventional and one classical antipsychotic and at least five episodes of auditory hallucinations per day during the past month. All patients were on stable antipsychotic medication for at least 3 weeks before and throughout the rTMS treatment. Exclusion criteria included significant neurological illness or head trauma, unstable medical condition, current alcohol or drug abuse, pregnancy, or MRI contraindication. A total of 32 patients was enrolled in the study. In the meantime, 35 matched healthy controls were recruited; their inclusive criteria were without any history of Axis I disorders or a first-degree relative with a psychotic illness. All participants provided their informed consent before this study. The study trial was registered in the Chinese Clinical Trial Register^1^ (registration number: ChiCTR2100041876) and approved by the Medical Ethics Committee of Xijing Hospital.

### rTMS Protocol

rTMS treatment was provided for 15 min per day on 15 successive days. The treatment was administered at 1 Hz and 110% of the resting motor threshold (RMT). RMT was determined before the stimulation session according to the standard method (Hoffman et al., 2003), which produced a motor evoked potential of no less than 50 mV in ten trials delivered. The stimulation site was targeted at the TPJ area referenced to the TP3 according to the International 10–20 EEG electrode position system. The stimulus pulse was delivered in sessions by YRD CCY-I magnetic stimulator (YIRUIDE Inc., Wuhan, China) with the figure of 8 coil, at one pulse per second for 10 s, with 5 s interval, consisted of 600 pluses with 60 trains.

### Clinical and Neurocognitive Investigation

The clinical symptoms were assessed with the Positive and Negative Scale (PANSS; Kay et al., 1987) and the Auditory Hallucination Rating Scale (AHRS; Hoffman et al., 2005). Neurocognitive assessments were completed using the Chinese version of MATRICS consensus cognitive battery (MCCB; Shi et al., 2015). The Chinese version of MCCB includes nine standardized subtests which reflect seven cognitive domains, including speed of processing (SOP), attention and vigilance (AV), WM, VerbLearn, VisLearn, reasoning and problem-solving (RPS), and social cognition (SC). Experienced psychiatrists executed all assessments at baseline and the end of the rTMS treatment.

### Imaging Data Acquire and Preprocessing

The patients underwent scanning within 48 h before the commencement of rTMS treatment and on the day following the end of the treatment course. The healthy controls were only scanned at baseline. Imaging data were acquired on a 3.0 Tesla MRI system with a standard 8-channel head coil (GE Medical Systems, Milwaukee, WI, United States). Earplugs and foam pads were used to minimize scanner noise and head motion. Participants were instructed to close their eyes and remain awake during the scan state. Functional images were acquired using a gradient echo-planar imaging (EPI) sequence (repetition time, 2,000 ms; echo time, 40 ms; field of view, 240 mm × 240 mm flip angle, 90°; matrix, 64 × 64; slice thickness, 3.5 mm; 45 axial slices no gap. A total of 210 volumes were collected for a total scan time of 420 s. Subsequently, high-resolution 3D T1-weighted anatomical images were acquired with an MPRAGE sequence (repetition time, 8.1 ms; echo time, 3.2 ms; field of view, 240 mm × 240 mm; flip angle, 10°; matrix, 256 × 256; slice thickness, 1 mm; and 176 slices sagittal slices).

All images were visually inspected for major artifacts prior to preprocessing. Functional Image data preprocessing was performed using Data Processing Assistant for Resting-State fMRI (DPABI; Yan et al., 2016). The initial ten scan volumes were discarded for steady-state magnetization, and subsequent images were corrected for temporal differences by slice timing and head motion by alignment. The resulting functional images were spatially normalized to the standard space of the Montreal Neurological Institute (MNI) using an optimum affine transformation and non-linear deformations (Ashburner and Friston, 1999), and then resampled to 3 mm × 3 mm × 3 mm isotropic voxels. Nuisance signals, including those from Friston motion parameters, white matter signals, cerebrospinal fluid signals, and mean global signals, were regressed out. Then all the functional images were smoothed with a 6-mm full-width at half-maximum (FWHM) Gaussian filter. Time series linear detrending was conducted to remove low-frequency drifts and high-frequency physiological noise.

Preprocessing of T1-weighted was used by SPM 8 (Statistical Parametric Mapping, Institute of Neurology, London, United Kingdom) accessed through the VBM 8 toolbox.^2^ Images were bias-corrected and tissue-classified into gray matter, white matter, and cerebrospinal fluid with the volume probability maps. The gray matter images were registered through the DARTEL approach and normalized to MNI space. The resulting images were modulated and smoothed using an 8 mm FWHM Gaussian filter.

### ALFF and VBM Analysis

The ALFF analysis was carried out using the DPABI toolkit. ALFF was calculated by obtaining the square root of the signal across 0.01--0.1 Hz for each voxel of the whole brain. For standardization purposes and reducing the influence of individual variation across participants, ALFF was further normalized by dividing the mean within the brain ALFF value for each participant. This created a standardized whole-brain ALFF map. VBM analysis was performed by SPM 8 package,^3^ the modulated and smoothed gray matter imagines were entered into the second level module to compare the GMV differences between the patient and control group.

### Statistical Analysis

Demographic, clinical, and neurocognitive results were processed in SPSS 20.0 (IBM, Armonk, New York, United States). Two-sample *t*-test and chi-square tests were used to examine group differences for continuous and categorical variables. In addition, independent sample *t*-tests were used to compare ALFF and GMV differences at baseline between patient group and control groups, and paired sample *t*-tests were used to compare ALFF and GMV alternations between baseline and post-rTMS treatment in the patient group. In addition, multiple linear regression analysis was used to explore whether the baseline ALFF and GMV values could be associated with the clinical symptoms and neurocognitive measurements after rTMS treatment in patients.

## Results

### Demographic, Clinical, and Neurocognitive Outcomes

The demographic and clinical characteristics that were available for participants are displayed in Table 1. Two patients and two controls were excluded because of excessive head motion artifacts (head motion exceeded 3 mm or rotation that exceeded 2.5°). The remaining 30 patients and 33 controls were included in the further analysis. The average age of patients included in the present study was 30.30 years old (SD = 4.46, ranging from 18 to 47 years). Seventeen patients (56.76%) were male. The average illness duration was 21.36 months (SD = 4.89, ranged from 10 to 38 months). There were no significant differences between patients and controls in age (*t* = 0.954, *p* = 0.345), sex (χ^2^ = 0.101, *p* = 0.751), and education (*t* = 1.708, *p* = 0.094).

**TABLE 1:** Demographic and clinical characteristics for participants.

Variable	Patients (*n* = 30)		Controls (*n* = 33)	*t* (χ^2^)	*p*
	Baseline	Post-rTMS			
Age (year)	30.30 ± 4.46	–	32.03 ± 7.31	0.954	0.345
Sex (female/male)	17 (13)	–	20 (13)	0.101	0.751
Education (year)	13.20 ± 2.67	–	12.09 ± 2.04	1.708	0.094
Duration of illness (months)	21.36 ± 4.89	–	–	–	–
CPED (mg/day)^a^	584.8 ± 152.39	–	–	–	–

*^*a*^CPED, Chlorpromazine equivalent doses (Woods, 2003).*

After rTMS treatment, the total scores (79.85 ± 10.55 vs. 67.50 ± 7.98), positive symptom scores (19.65 ± 4.59 vs. 14.45 ± 2.80), and general symptom scores (40.35 ± 6.65 vs. 35.20 ± 5.54) in PANSS, and AHRS scores (27.45 ± 6.14 vs. 13.75 ± 7.07) significantly decreased relative to pre-treatment in patients (all *p* < 0.01).

**FIGURE 1:**
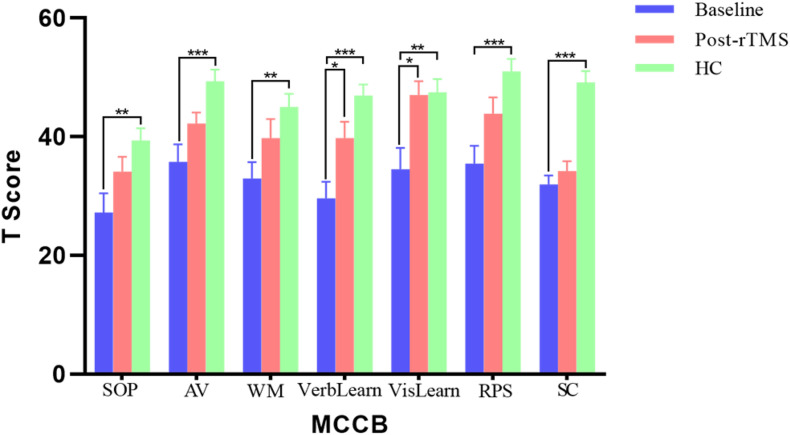
The results of MATRICS consensus cognitive battery (MCCB) comparisons between patients (baseline and post treatment) and controls. *p < 0.05, **p < 0.01, and ***p < 0.001.

In addition, the patients showed neurocognitive impairments in all seven cognitive domains of MCCB compared to the health controls (all *p* < 0.01) with the multiple comparison corrections using the false discover rate (FDR) approach (Figure 1). Moreover, after the rTMS treatment, there were significant improvements in several cognitive domains (FDR correction, *p* < 0.05) in patients, including verbal learning (29.60 ± 12.60 vs. 39.60 ± 12.24, *p* = 0.035) and visual learning (34.55 ± 15.95 vs. 47.00 ± 10.54, *p* = 0.031).

### Neuroimaging Comparisons at Baseline Between the Patients and Controls

**FIGURE 2:**
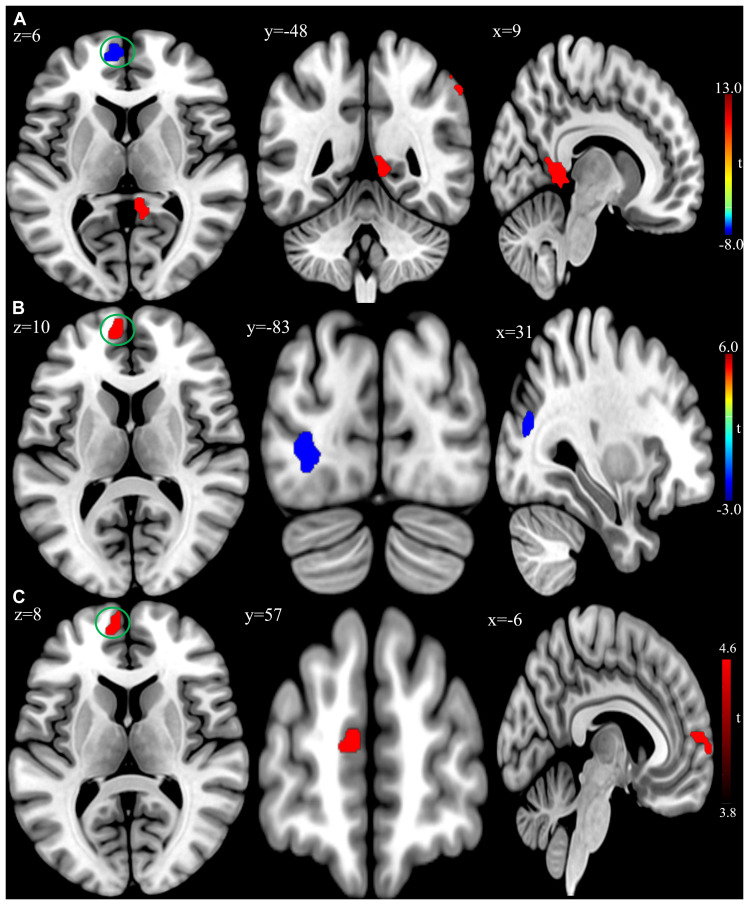
**(A)** Brain regions showing significant differences of amplitude of low-frequency fluctuation (ALFF) between patient group and healthy control group at baseline. **(B)** Brain regions showing significant differences of ALFF between the posttreatment and baseline in the patient group. **(C)** Conjunction analysis maps of ALFF differences [(baseline vs. controls) ∩ (posttreatment vs. baseline)]. The warm color denoted the region where ALFF is higher, and the cool color denotes the region where ALFF is lower. The color circle denoted the overlapped region of ALFF differences.

At the baseline, ALFF analysis showed that patients had lower ALFF in the left superior frontal gyrus and higher ALFF in the right inferior parietal lobule and right precuneus compared to the controls (Table 2 and Figure 2A). The cluster threshold was set at *p* < 0.05 and voxel-level at *p* < 0.01 (size > 30) with FDR correction.

**TABLE 2:** Amplitude of low-frequency fluctuation differences between the patient and control group at baseline.

Brain region	Broadmann	MNI coordinate			Cluster size	*T*
		*x*	*y*	*z*		
Left superior frontal gyrus (medial)	10	−9	60	9	106	−8.34
Right inferior parietal lobule	40	54	−57	48	51	8.49
Right precuneus	27	12	−39	0	85	12.68

**FIGURE 3:**
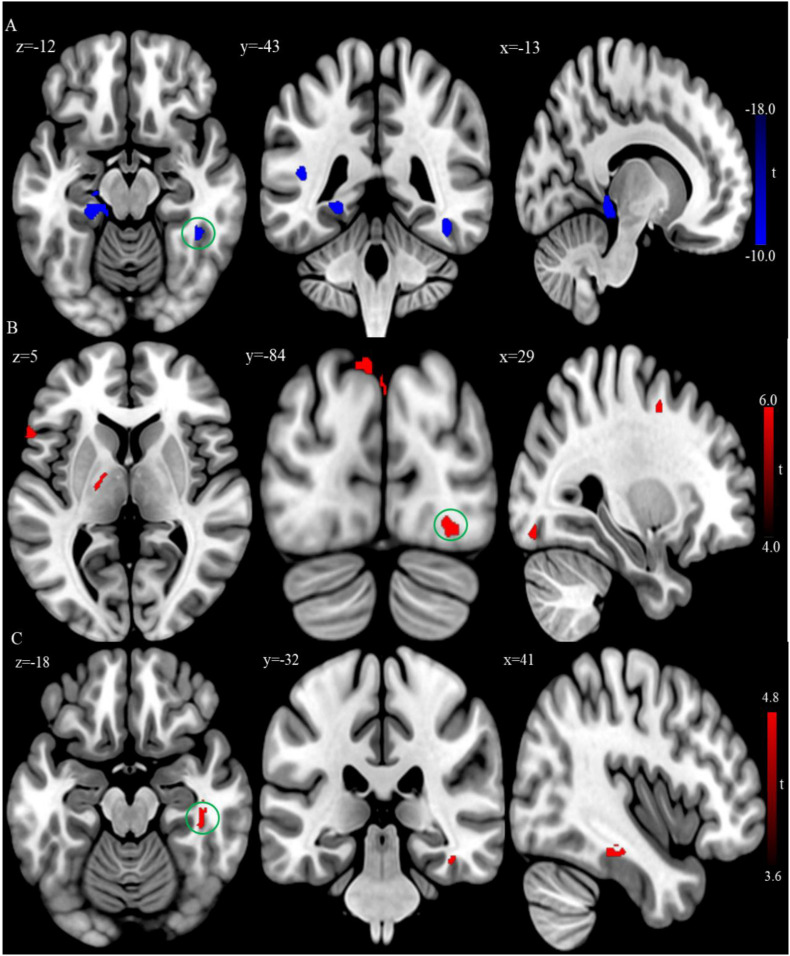
**(A)** Brain regions showing significant differences of gray matter volume (GMV) between the patient group and healthy control group at baseline. **(B)** Brain regions showing significant difference of GMV between the posttreatment and baseline in the patient group. **(C)** Conjunction analysis maps of GMV differences [(baseline vs. controls) ∩ (posttreatment vs. baseline)]. The warm color denotes the region where the GMV is larger, and the cool color denotes the region where the GMV is smaller. The color circle denoted the overlapped region of GMV differences.

In addition, VBM analysis showed that the GMV of the bilateral superior temporal gyrus was significantly larger, while the GMV of the left posterior cingulate gyrus and parahippocampus gyrus were significantly smaller in the patient group relative to the control group at baseline (Table 3 and Figure 3A; FDR correction, cluster-level *p* < 0.05, voxel-level *p* < 0.01, size > 30).

**TABLE 3:** Gray matter volume differences between patient and control group at baseline.

Brain region	Broadmann	MNI coordinates			Cluster size	*T*
		*x*	*y*	*z*		
Left inferior temporal gyrus	38	−48	−3	−35	217	−11.85
Right inferior temporal gyrus	38	35	−14	−41	372	−13.08
Left inferior frontal gyrus	47	−24	36	2	231	−14.97
Left posterior cingulate cortex	26	−3	−38	15	250	−16.41
Left thalamus	−	−17	−8	8	182	−15.71

### Neuroimaging Comparisons Between Posttreatments and Baseline in Patients

Compared to baseline, the patients with rTMS treatment showed higher ALFF in the left superior frontal gyrus, while lower ALFF in the left inferior occipital gyrus and right middle occipital gyrus (FDR correction, cluster-level *p* < 0.05, voxel-level *p* < 0.01, size > 30; Table 4 and Figure 2B). Additionally, conjunction analysis (Nichols et al., 2005) was used to identity whether there are areas presenting jointly significant differences of ALFF for both the baseline and post-treatment comparisons. The ALFF of the left superior frontal gyrus was found to be jointly and significantly higher in posttreatment compared to baseline in patient group (Figure 2C).

**TABLE 4:** Amplitude of low-frequency fluctuation differences between the post treatment and baseline in the patient group.

Brain region	Broadmann	MNI coordinates			Cluster size	*T*
		*x*	*y*	*z*		
Left superior frontal gyrus (medial)	8	−9	57	12	75	5.08
Left middle occipital gyrus	19	−27	−84	3	134	−3.94
Right middle occipital gyrus	19	30	−75	18	105	−5.39

After rTMS treatment, the patient group had larger GMV in the bilateral inferior temporal cortex, left inferior parietal lobule, and superior occipital cortex relative to baseline (FDR correction, cluster-level *p* < 0.05, voxel-level *p* < 0.01, size > 30; Table 5 and Figure 3B). Also, the conjunction analysis revealed that the GMV of the right inferior temporal gyrus was jointly larger in patients with rTMS than baseline (Figure 3C).

**TABLE 5:** Gray matter volume differences between the posttreatment and baseline in the patient group.

Brain region	Broadmann	MNI coordinate			Cluster size	*T*
		*x*	*y*	*z*		
Left inferior frontal gyrus	47	−60	20	3	53	4.84
Right inferior frontal gyrus	47	45	51	−21	67	4.42
Right inferior temporal gyrus	20	50	−30	−20	199	5.50
Left middle occipital gyrus	18	−29	−87	45	553	5.70
Right middle occipital gyrus	19	−14	−89	47	65	5.04
Left thalamus	−	−18	−11	6	150	4.16

### Linear Regression Analysis in Patient Group

For the patient group, multiple linear regression analyses were performed with the baseline ALFF and GMV values of the significant brain regions as independent variables and clinical as well as neurocognitive scores after rTMS treatment as dependent variables, obtaining the relationship between the brain metrices and clinical symptoms or neurocognitive performances (age, sex, education, and illness of disease as covariates; Table 6). The regression analysis revealed a significant positive association between baseline ALFF value of the right inferior parietal lobule and the severity of positive symptoms after rTMS treatment (*B* = 0.507, *p* = 0.043). In addition, the multiple regression showed that the baseline GMV values of the left and right superior temporal gyrus were associated with the verbal learning performance (*B* = 0.748, *p* = 0.035; *B* = 0.837, *p* = 0.025) and visual learning performance (*B* = 0.738, *p* = 0.036; *B* = 0.731, *p* = 0.045) after rTMS treatment.

**TABLE 6:** Results of regression analysis at baseline brain metrics on responses to repetitive transcranial magnetic stimulation in the patient group.

	AHRS		Positive symptoms		Verbal learning		Visual learning	
	*B*	*p*	*B*	*p*	*B*	*p*	*B*	*p*
**ALFF**								
L SFGmed	−0.036	0.899	0.027	0.924	0.244	0.581	−0.326	0.460
R IPL	−0.171	0.472	0.507	0.043*	0.435	0.242	−0.521	0.163
R PCUN	0.104	0.709	0.234	0.398	0.100	0.815	0.054	0.898
**GMV**								
L STG	−0.053	0.804	−0.374	0.087	0.748	0.035*	0.738	0.036*
R STG	−0.226	0.318	−0.130	0.555	0.837	0.025*	0.731	0.045*
L IFG	0.051	0.824	−0.152	0.500	−0.278	0.431	0.769	0.040*
L PCC	−0.090	0.747	−0.122	0.656	−0.330	0.444	0.381	0.375
L Thala	0.293	0.209	−0.025	0.917	−0.063	0.792	−0.035	0.882

*^∗^*p* < 0.05. L, left; R, right; SFG, superior frontal gyrus; Med, medial; IPL, inferior parietal lobule; PCUN, precuneus; STG, superior temporal gyrus; IFG, inferior frontal gyrus; PCC, posterior cingulate cortex; Thala, thalamus.*

## Discussion

The present study investigated the effect of low-frequency rTMS over the left TPJ area for treating schizophrenia patients with AVH and the following brain functional and structural changes (e.g., ALFF and GMV). The results showed that the positive symptoms, including AVH, were reduced, and certain neurocognitive functions were improved after the rTMS treatment in patients. The results were consistent with the previous report (Aleman et al., 2007) and confirmed that low-frequency rTMS was efficacious in treating schizophrenia patients with AVH. Moreover, rTMS treatment induced ALFF and GMV changes in certain brain regions, and the baseline brain metrics were associated with the positive symptoms as well as neurocognitive performances after rTMS treatment in patient group.

After rTMS treatment, some brain functional and structural changes were observed by resting-state fMRI data analysis in patients. Among these results, the following seem to be interesting. Firstly, ALFF was jointly higher in the left superior frontal gyrus (medial part, BA10) after rTMS treatment. ALFF is thought to reflect spontaneous low-frequency fluctuation of neural activity in a particular voxel. The medial part of superior frontal gyrus (MSFG) is a subdivision of superior frontal cortex (Li et al., 2013) and implicated in the pathophysiology of schizophrenia (Pomarol-Clotet et al., 2010). The MSFG has been described to be a major hub of the default mode network, which is typically more active at rest (Buckner et al., 2008). While this area is hypoactive during the resting state in schizophrenia and associated with the severity of clinical symptoms (Williams et al., 2004; Park et al., 2009; Berger et al., 2018). Then it was possible to observe lower spontaneous activity (e.g., ALFF) of this area in the schizophrenia patients with AVH. Besides, the left MSFG has been suggested to be a part of the complex cortical network related to language processing (Li et al., 2009; Rapp and Steinhäuser, 2013). Patients with schizophrenia have significantly lower signal variations of this cortex compared to controls and could represent an underlying neural basis for impaired language communication and comprehension (Dollfus et al., 2008; Razafimandimby et al., 2016). And the MSFG receives projections from various cortical regions within the lobe (e.g., the superior temporal gyrus and temporal pole; Bachevalier et al., 1997) and could constitute an auditory language network (Reyes-Aguilar et al., 2018). Therefore, abnormality in the MSFG could be reversed through resetting local AVH-related circuits via rTMS treatment and manifested as higher AFLL of this area in patients.

It was worth that the patients had higher baseline ALFF of the right inferior parietal lobule compared to the healthy controls, which met the previous studies (Li et al., 2016; Liang et al., 2019), and were associated with the severity of positive symptoms after rTMS treatment. The inferior parietal lobule is an important part of the frontal-limbic-temporal-parietal network implicated in the schizophrenia disease process (Torrey, 2007), and its overactivity is associated with the presence of positive symptoms in schizophrenia patients (Yildiz et al., 2011). Hence the functional abnormality of the inferior parietal lobule seems to be involved in the clinical symptoms of psychosis.

Secondly, the baseline GMV of the temporal regions was jointly significantly larger in patients after rTMS treatment. AVH arises from a defective self-monitoring of inner speech (Seal et al., 2004; Ditman and Kuperberg, 2005). Self-monitoring of inner speech is associated with activations in the temporal cortex (Frith and Done, 1988). Schizophrenia patients with AVH have attenuated activation in regions implicated in the routine monitoring of inner speech (e.g., temporal cortex; McGuire et al., 1995; Shergill et al., 2000b). The structural abnormalities of the temporal cortex (e.g., reduced GMV) are also frequently reported in schizophrenia patients with AVH (Rajarethinam et al., 2000; Job et al., 2002; Onitsuka et al., 2004). One possible explanation for larger GMV in the right inferior temporal gyrus post-treatment is that rTMS directly stimulating over the left TPJ area causes brain structural changes on adjacent regions such as the inferior temporal gyrus because of intrinsic connections within these regions (Scheinost et al., 2012).

Another interesting finding was that ALFF was lower in the middle occipital gyrus after the rTMS treatment in patients. Results on ALFF changes in the middle occipital gyrus for schizophrenia were mixed (Gong et al., 2020). Some studies reported a decreased ALFF of the middle occipital gyrus in schizophrenia patients compared to healthy controls (Yu et al., 2014), while others indicated an increased ALFF in the same area (Ren et al., 2013). Although we did not see any change of ALFF in the area at baseline, we speculated that low-frequency rTMS might produce an inhibitory effect on underlying neuronal populations involved in the target site (e.g., TPJ area). The left TPJ area is implicated in cognitive control (Mayer et al., 2004) and connected with the right middle occipital gyrus (Wu et al., 2015), so the inhibition of low-frequency rTMS over the left TPJ would induce connectivity-mediated brain hemodynamic changes and result in the deactivation of the relevant brain areas (e.g., the right middle occipital cortex).

Additionally, this study demonstrated that widespread cognitive impairments existed in schizophrenia patients with AVH in all seven cognitive domains of MCCB and agreed with the previously published studies, which reported that patients with schizophrenia had widespread cognitive deficits (Schuepbach et al., 2002; Chen et al., 2021). Furthermore, we found that certain cognitive domains, such as verbal learning and visual learning, were improved after the rTMS treatment for patients. The results suggest that low-frequency rTMS could contribute to improvements of certain cognitive domains, similar to the high-frequency rTMS protocol applied in schizophrenia (Mogg et al., 2007; Demirtas-Tatlidede et al., 2010; Oh and Kim, 2011), which reported better performance in verbal learning and visual learning in rTMS group.

Specifically, the smaller volume has been identified in the superior temporal gyrus in patients with schizophrenia (Bandeira et al., 2020) and is not due to the effect of medication (Takahashi et al., 2010), which represents a vulnerability marker of psychosis (Borgwardt et al., 2007). In addition to manifesting in psychotic symptoms such as hallucinations (Barta et al., 1990), the superior temporal gyrus is implicated in cognitive functions such as verbal memory (Grasby et al., 1993). Lesion of this cortex impairs verbal memory performance (Takayama et al., 2004). In addition, the superior temporal gyrus is involved in visuospatial processing (e.g., visual search and spatial perception; Karnath et al., 2001; Ellison et al., 2004; Gharabaghi et al., 2006). Therefore, we observed that the baseline GMV of the superior temporal gyrus could be associated with certain cognitive functions after rTMS treatment in patient group, including verbal learning and visual learning, and suggested that the brain structural metric of this cortex is related to cognitive performance (Achiron et al., 2013). The GMV of the left inferior frontal gyrus could be correlated with the patients’ the visual learning performance after rTMS treatment, which was consistent with the previous report that this cortex is involved in visual learning and memory (Grèzes and Decety, 2002; Manjaly et al., 2005), and damage to this cortex results in impairment of visual task performance (Husain and Kennard, 1996). These results suggested the GMV of the superior temporal cortex and the inferior frontal cortex were important markers of cognitive impairments in schizophrenia and specifically related to verbal learning and visual learning, as these brain areas are known to be associated with speech comprehension and visuospatial processing.

Some limitations of the present study should be considered. One major limitation of this study is the lack of placebo sham-stimulation control for low-frequency rTMS, which checked our conclusion on the efficacy of the stimulation paradigm. Future research should use the sham-stimulation control to reduce the possible placebo effect. Another potential limitation includes the control participants were only scanned once, which produced possible confusion on the generality of the results. Future research should preferably use the standard protocol to improve the reliability of the results.

## Conclusion

The present study investigated the alternations in brain function and structure induced by low-frequency rTMS over the left TPJ area in schizophrenia patients with AVH using resting-state fMRI data. Our findings showed that the rTMS treatment was efficacious for reducing positive symptoms and improving cognitive functions in patients. And the rTMS treatment could lead to specific brain functional and structural alternations. The baseline functional fluctuation (e.g., ALFF) and structural integrity (e.g., GMV) at certain brain areas could be associated with the positive symptoms and neurocognitive functions in patients with rTMS treatment.

## Data Availability Statement

The raw data supporting the conclusion of this article will be made available by the authors, without undue reservation.

## Ethics Statement

The studies involving human participants were reviewed and approved by the Medical Ethics Committee of Xijing Hospital, Fourth Military Medical University. The patients/participants provided their written informed consent to participate in this study.

## Author Contributions

YJX and MZG: conceptualization, methodology and software, and writing. YJX, MZG, and ZHW: validation and formal analysis. YJX, ZHW, and ZJM: data curation. HNW and HY: supervision. PF, HNW, and HY: funding acquisition. All authors have read and agreed to the published version of the manuscript.

## Conflict of Interest

The authors declare that the research was conducted in the absence of any commercial or financial relationships that could be construed as a potential conflict of interest.

## Publisher’s Note

All claims expressed in this article are solely those of the authors and do not necessarily represent those of their affiliated organizations, or those of the publisher, the editors and the reviewers. Any product that may be evaluated in this article, or claim that may be made by its manufacturer, is not guaranteed or endorsed by the publisher.

## Footnotes

1
http://www.chictr.org.cn

2
http://dbm.neuro.uni-jena.de/vbm/

3
http://www.fil.ion.ucl.ac.uk/spm/software/spm8/
