# Corrigendum: rTMS induces brain functional and structural alternations in schizophrenia patient with auditory verbal hallucination

**DOI:** 10.3389/fnins.2022.1118304

**Published:** 2023-01-17

**Authors:** Yuanjun Xie, Muzhen Guan, Zhongheng Wang, Zhujing Ma, Huaning Wang, Peng Fang, Hong Yin

**Affiliations:** ^1^Department of Radiology, Xijing Hospital, Fourth Military Medical University, Xi'an, China; ^2^Department of Mental Health, Xi'an Medical University, Xi'an, China; ^3^Department of Psychiatry, Xijing Hospital, Fourth Military Medical University, Xi'an, China; ^4^Department of Clinical Psychology, School of Medical Psychology, Fourth Military Medical University, Xi'an, China; ^5^Department of Military Medical Psychology, School of Medical Psychology, Fourth Military Medical University, Xi'an, China

**Keywords:** schizophrenia, auditory verbal hallucination, transcranial magnetic stimulation, MCCB, amplitude of low-frequency fluctuation, voxel-based morphometry

In the published article, there was an error in Figure 2 as published. The bottom of Figure 2 was slightly cropped. The revised Figure 2 appears below.

**Figure 2:**
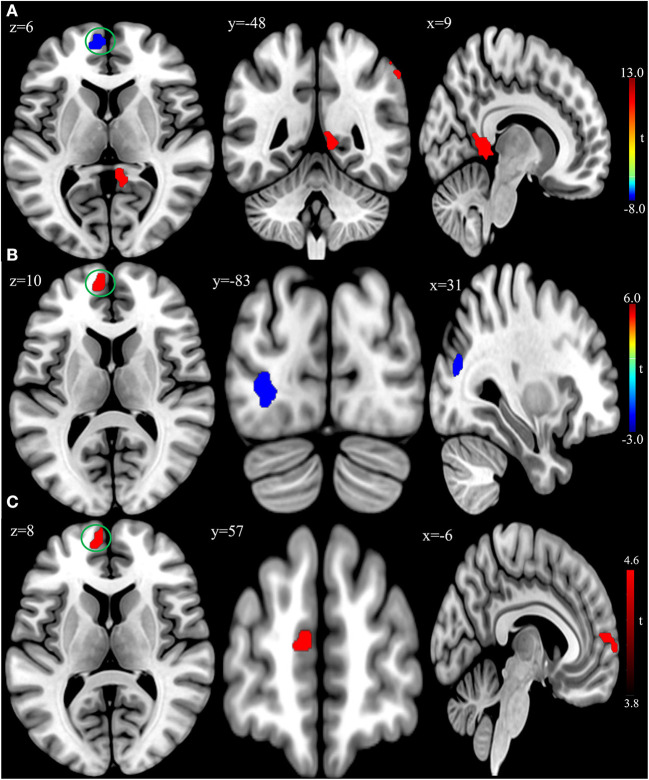
**(A)** Brain regions showing significant differences of amplitude of low-frequency fluctuation (ALFF) between patient group and healthy control group at baseline. **(B)** Brain regions showing significant differences of ALFF between the posttreatment and baseline in the patient group. **(C)** Conjunction analysis maps of ALFF differences [(baseline vs. controls) n (posttreatment vs. baseline)]. The warm color denoted the region where ALFF is higher, and the cool color denotes the region where ALFF is lower. The color circle denoted the overlapped region of ALFF differences.

In the published article, there was also an error in **Materials and Methods**, *Imaging Data Acquire and Preprocessing*, Paragraph 1. The repetition time and echo time were incorrectly given as “repetition time, 1,900 ms; echo time, 2.26 ms” but should be “repetition time, 8.1 ms; echo time, 3.2 ms”. The corrected paragraph appears below:

“The patients underwent scanning within 48 h before the commencement of rTMS treatment and on the day following the end of the treatment course. The healthy controls were only scanned at baseline. Imaging data were acquired on a 3.0 Tesla MRI system with a standard 8-channel head coil (GE Medical Systems, Milwaukee, WI, United States). Earplugs and foam pads were used to minimize scanner noise and head motion. Participants were instructed to close their eyes and remain awake during the scan state. Functional images were acquired using a gradient echo-planar imaging (EPI) sequence (repetition time, 2,000 ms; echo time, 40 ms; field of view, 240 mm × 240 mm; flip angle, 90°; matrix, 64 × 64; slice thickness, 3.5 mm; 45 axial slices no gap. A total of 210 volumes were collected for a total scan time of 420 s. Subsequently, high-resolution 3D T1-weighted anatomical images were acquired with an MPRAGE sequence (repetition time, 8.1 ms; echo time, 3.2 ms; field of view, 240 mm × 240 mm; flip angle, 10°; matrix, 256 × 256; slice thickness, 1 mm; and 176 slices sagittal slices)”.

The authors apologize for these errors and state that this does not change the scientific conclusions of the article in any way. The original article has been updated.

